# Supplementary material for: Convergent functional effects of antidepressants in major depressive disorder: a neuroimaging meta-analysis
Source: Mol Psychiatry. 2024 Oct 15;30(2):736–51. doi: 10.1038/s41380-024-02780-6 (PMC11746144; doi:10.1038/s41380-024-02780-6)
Supplement: Supplementary file 1 — Supplementary Information [file 41380_2024_2780_MOESM1_ESM.pdf]

Supplementary Information

Convergent functional effects of antidepressants in major depressive disorder: a neuroimaging meta-analysis

Amin Saberi[1,2,3], Amir Ebneabbasi[4,5], Sama Rahimi[1,2,6], Sara Sarebannejad[7], Zumrut Duygu Sen[8,9,10,11], Heiko Graf[12], Martin Walter[8,9,10,11,13], Christian Sorg[14,15,16], Julia A. Camilleri[1,2], Angela R. Laird[17], Peter T. Fox[18], Sofie L. Valk[1,2,3], Simon B. Eickhoff[1,2], Masoud Tahmasian[1,2,19]

[1] Institute of Neurosciences and Medicine (INM-7), Research Centre Jülich, Jülich, Germany.
[2] Institute of Systems Neuroscience, Medical Faculty and University Hospital Düsseldorf, Heinrich Heine University Düsseldorf, Düsseldorf, Germany.
[3] Max Planck Institute for Human Cognitive and Brain Sciences, Leipzig, Germany
[4] Department of Clinical Neurosciences, University of Cambridge, Biomedical Campus, Cambridge CB2 0QQ, UK.
[5] Cambridge University Hospitals NHS Trust, Cambridge CB2 0SZ, UK.
[6] Neuroscience Center, Goethe University, Frankfurt, Hessen, Germany.
[7] Kavli Institute for Systems Neuroscience, Norwegian University of Science and Technology, Trondheim, Norway.
[8] Department of Psychiatry and Psychotherapy, University Hospital Jena, Jena, Germany.
[9] Clinical Affective Neuroimaging Laboratory (CANLAB), Magdeburg, Germany.
[10] Department of Psychiatry and Psychotherapy, University Tübingen, Tübingen, Germany.
[11] German Center for Mental Health, Halle-Jena-Magdeburg, DZP, Germany.
[12] Department of Psychiatry and Psychotherapy III, University of Ulm, Ulm, Germany.
[13] Leibniz Institute for Neurobiology, Brenneckestr. 6, 39118 Magdeburg, Germany.
[14] TUM-Neuroimaging Center, School of Medicine and Healthy, Technical University Munich, Munich, Germany.
[15] Department of Neuroradiology,School of Medicine and Healthy, Technical University Munich, Munich, Germany.
[16] Department of Psychiatry, School of Medicine and Healthy, Technical University Munich, Munich, Germany.
[17] Department of Physics, Florida International University, Miami, FL, USA.
[18] Research Imaging Institute, University of Texas Health Science Center at San Antonio, San Antonio, TX, USA.
[19] Department of Nuclear Medicine, University Hospital and Medical Faculty, University of Cologne, Cologne, Germany.

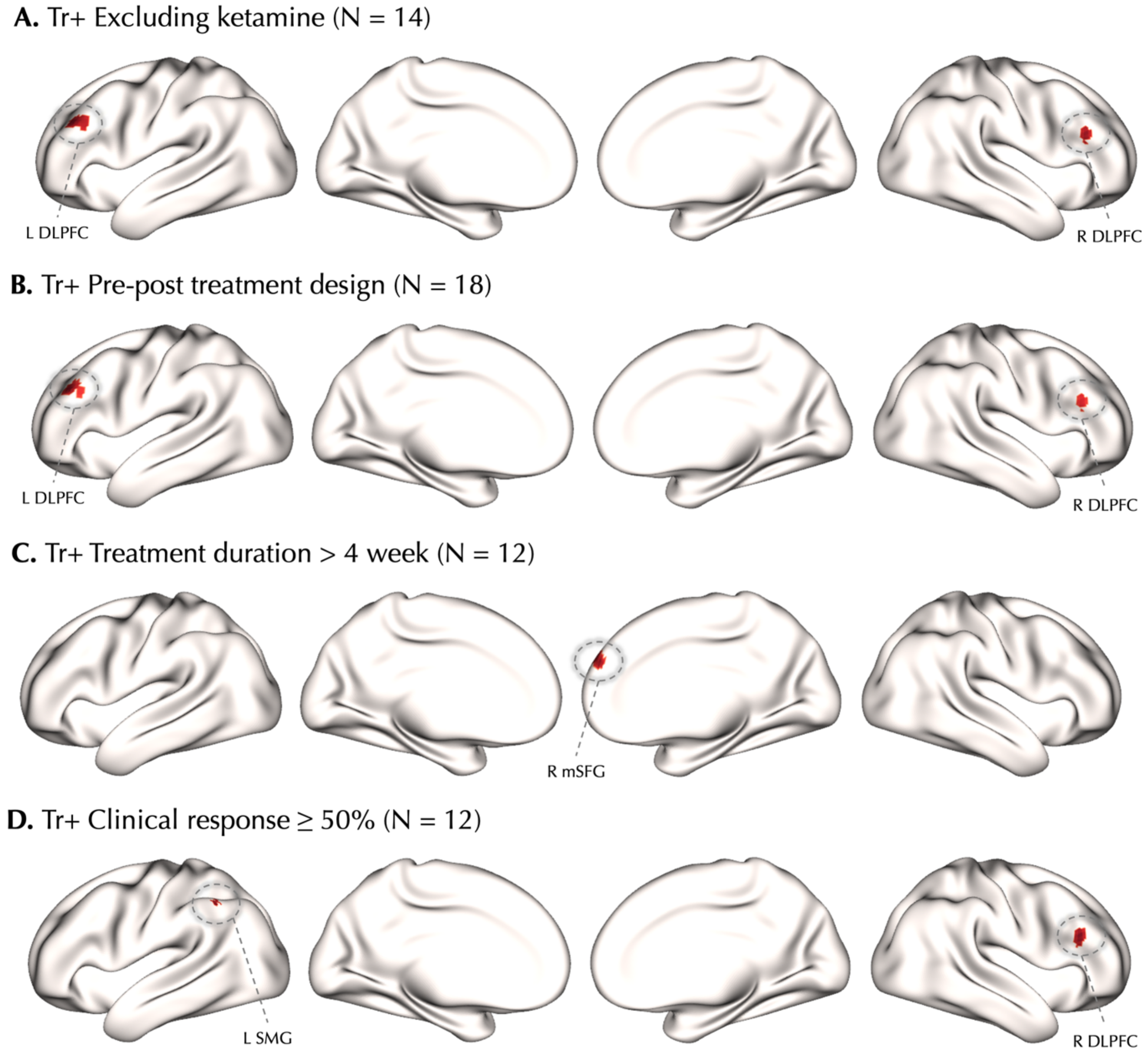

Fig S1. Subgroup ALE meta-analyses of Treated > Untreated experiments. The ALE significant clusters of convergence in the different subgroups of Treated > Untreated (Tr+) experiments are shown. N represents the number of experiments included in each ALE meta-analysis.

L: left, R: right, DLPFC: dorsolateral prefrontal cortex, mSFG: medial superior frontal gyrus, SMG: supramarginal gyrus

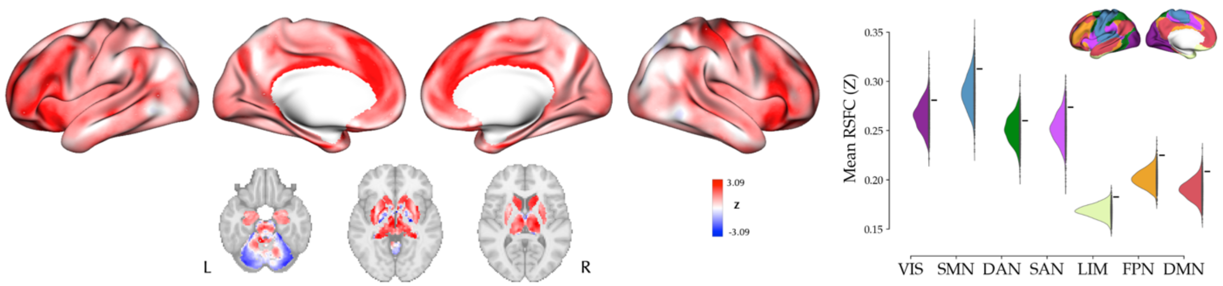

Fig. S2. Convergent connectivity mapping of antidepressant effects without weighting by sample size. *left*: The cortical and subcortical map represent convergent connectivity map of the foci from all experiments in which the convergent connectivity across experiments is averaged without weighting by their sample sizes. *right*: Mean resting state functional connectivity (RSFC) of the observed foci across canonical resting state networks (denoted by "-") compared against null mean values calculated based on 1000 permutations of randomly selected foci (half-violin plots). None of the networks showed significantly more extreme observed mean RSFC than the null distribution in a two-tailed test and after false discovery rate adjustment at 5%.

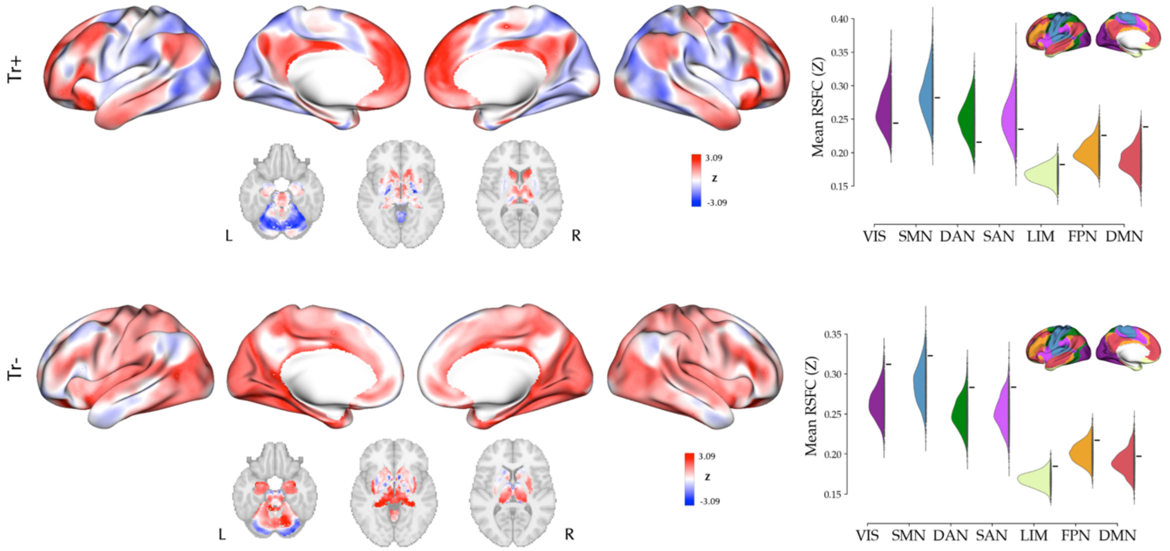

Fig. S3. Convergent connectivity mapping of antidepressant effects across contrasts. *left:* The cortical and subcortical maps represent convergent connectivity maps of the Treated > Untreated (Tr+; *top*) and Untreated > Treated (Tr-; *bottom*) experiments. *right:* Mean resting state functional connectivity (RSFC) of the observed foci across canonical resting state networks (denoted by "-") compared against null mean values calculated based on 1000 permutations of randomly selected foci (half-violin plots) for the Tr+ (*top*) and Tr- (*bottom*) experiments. In both contrasts none of the networks showed significantly more extreme observed mean RSFC than the null distribution in a two-tailed test and after false discovery rate adjustment at 5%.

VIS: visual network, SMN: somatomotor network, DAN: dorsal attention network, SAN: salience network, LIM: limbic network, FPN: frontoparietal network, DMN: default mode network.

Table S1. Search keywords.

	Search Terms
#1	MDD OR "Major Depressive Disorder" OR "Unipolar Depressive Disorder" OR Depress*
#2	(Antidepres*) OR (SSRI* OR SRI OR SRIs OR "Selective Serotonin Reuptake Inhibitor*" OR "Serotonin Uptake Inhibitor*" OR Dapoxetine OR Citalopram OR Escitalopram OR Fluoxetine OR Fluvoxamine OR Mirtazapine OR Paroxetine OR Sertraline OR Vilazodone OR Zimelidin) OR (SNRI* OR "Serotonin Norepinephrine Uptake Inhibitor*" OR Bicifadine OR Venlafaxin OR Desvenlafaxin OR Duloxetine OR "Duloxetine Hydrochloride" OR Milnacipran OR Levomilnacipran OR Levomepromazine OR Sibutramine OR Bicifadine OR mianserin) OR ("Monoamine Oxidase Inhibitor*" OR MAOI* OR Isocarboxazid OR Phenelizine OR Tranylcypromine OR Selegiline OR Clorgyline OR Moclobemide OR Brofaromine) OR (Tricyclic OR Tetracyclic OR TCA OR TCAs OR Imipramine OR Desipramine OR Trimipramine OR Nortriptyline OR Amitriptyline OR Protriptyline OR Amoxapine OR Doxepin OR Maprotiline OR Clomipramine OR amineptine OR butriptyline OR Demexiptiline OR dibenzepin OR dothiepin OR lofepramine OR melitracen OR noxiptiline OR opipramol OR protriptyline OR quinupramine OR tianeptine OR chlorpoxiten OR amersergide) OR ("NMDA Antagonist*" OR ketamine OR esketamine) OR ("selective noradrenaline reuptake inhibitor*" OR NaRI* OR Reboxetine) OR ("Atypical Antidepres*" OR Mirtazapine OR Nefazodone OR Trazodone OR L-Tryptophan OR agomelatine OR vortioxetine OR Bupropion OR Quetiapine)
#3	fMRI OR "Functional magnetic resonance imaging" OR "functional MRI" OR "resting state" OR "resting-state" OR "gray matter" OR "grey matter" OR "regional cerebral blood flow" OR PET OR "positron emission tomography" OR SPECT OR "Single-photon emission computed tomography" OR ASL OR "arterial spin labeling" OR ALFF OR "Amplitude of low frequency fluctuations" OR fALFF OR "Fractional amplitude of low-frequency fluctuations" OR ReHo OR "Regional Homogeneity" OR ICA OR "Independent Component Analysis" OR Graph
	#1 AND #2 AND #3

Table S2. Methodological details of studies included in the meta-analysis.

#	First Author, Year [a]	Field strength	Task	Task stimulus	Main softwares	Contrast	Multiple comparisons correction
**1**	Abdallah, C. G., 2017	3 T	Rest	-	FSL, AFNI	Pre- vs. post-treatment	3dClustSim
	Murrough, J. W., 2015	3 T	Emotion perception	Faces	SPM, AFNI		
**2**	Bremner, J. D., 2007	-	Emotional word pair retrieval	Words	SPM	Pre- vs. post-treatment	Uncorrected (k > 40, p < 0.005)
**3**	Carlson, P. J., 2013	-	Rest	-	SPM	Pre- vs. post-treatment	RFT
**4**	Cheng, Y., 2017	1.5 T	Rest	-	SPM, REST	Pre- vs. post-treatment	AlphaSim
**5**	Downey, D., 2016	3 T	Rest	-	SPM	Time x Group (treated vs placebo MDD)	cFWE
**6**	Fonzo, G. A., 2019	3 T	Emotional conflict	Faces and words	FSL, SPM, R (fmri package)	Time x Group (treated vs placebo MDD)	FDR
**7**	Frodl, T., 2011	3 T	Emotion recognition	Faces	SPM	Pre- vs. post-treatment	cFWE
**8**	Fu, C. H., 2004, 2007	1.5 T	Emotion recognition	Faces	In-house	Time x Group (treated MDD vs HC)	cFWE
**9**	Fu, C. H., 2015	3 T	Affective facial expressions / Emotional Stroop	Faces; Words	SPM	Pre- vs. post-treatment	cFWE
**10**	Gonzalez, S., 2020	3 T	Rest	-	FSL, ASLtoolbox	Pre- vs. post-treatment	FDR
**11**	Jiang, W., 2012	1.5 T	Emotion recognition	Faces	SPM	Pre- vs. post-treatment	Uncorrected
**12**	Joe, A.Y., 2006	-	Rest	-	SPM	Pre- vs. post-treatment	Uncorrected (k > 100 voxels, p < 0.005)
**13**	Keedwell, P., 2008	1.5 T	Emotion perception	Faces	Institute of Psychiatry	Early vs. late post-treatment	cFWE
**14**	Kennedy, S. H., 2001	-	Rest	-	SPM	Pre- vs. post-treatment	cFWE
**15**	Kohn, Y., 2008	-	Rest	-	SPM	Pre- vs. post-treatment	cFWE

**16**	Komulainen, E., 2018	3 T	Emotional word processing	Words	SPM	Treatment vs. placebo	FDR
	Komulainen, E., 2021	3 T	Listening to emotional narratives	Speech	Intersubject Correlation Toolbox, FSL	Treatment vs. placebo	FDR
**17**	Kraus, C., 2019	7 T	Cued painful stimulation	Symbols +/- Electrical stimulation	SPM	Treated acute MDD vs. untreated remitted MDD	Uncorrected
	Rütgen, M., 2019	7 T	Empathy for pain	Faces	SPM, AFNI, FSL	Pre- vs. post-treatment	cFWE
**18**	Li, C. T., 2016	-	Rest	-	SPM	Pre- vs. post-treatment	cFWE
**19**	Lopez-Sola, M., 2010	1.5 T	Painful stimulation	Auditory tone and heat	SPM	Time x Group (treated MDD vs HC)	Uncorrected
**20**	Mayberg, H. S., 2000	-	Rest	-	In-house	Pre- vs. post-treatment	Uncorrected
**21**	Reed, J. L., 2018	3 T	Dot probe task with emotional faces	Faces and dots	AFNI	Session (treatment vs. placebo) x Group (MDD vs. HC)	3dClustSim
	Reed, J. L., 2019		Emotion recognition	Faces			
**22**	Robertson, B., 2007	1.5 T	Emotional oddball	Pictures and shapes	SPM	Pre- vs. post-treatment	Uncorrected
**23**	Sankar, A., 2017	3 T	Sternberg (verbal working memory)	Letters	SPM	Pre- vs. post-treatment	cFWE
**24**	Sterpenich, V., 2019	3 T	Monetary incentive delay task / Emotional judgment	Pictures	SPM	Pre- vs. post-treatment	cFWE
**25**	Wagner, G., 2010	1.5 T	Stroop Color-Word	Words	SPM	Pre- vs. post-treatment	Uncorrected
**26**	Walsh, N. D., 2007	1.5 T	N-back	Letters	n.a.	Pre- vs. post-treatment	cFWE
**27**	Wang, L., 2014	3 T	Rest	-	DPARSF, REST	Pre- vs. post-treatment	AlphaSim
	Wang, L., 2017					Time x Group (MDD vs. HC)	
**28**	Wang, Y., 2012	3 T	Emotion recognition	Faces	SPM	Pre- vs. post-treatment	Uncorrected
**29**	Williams, R. J., 2021	3 T	Emotional face matching	Faces	Sandwich Estimator	Pre- vs. post-treatment	TFCE

					Toolbox, SPM		
**30**	Yin, Y., 2018	3 T	Rest	-	SPM	Pre- vs. post-treatment	AlphaSim

[a] Publications with overlapping samples are grouped together.

n.a.: not available, AFNI: Analysis of Functional NeuroImages, cFWE: family-wise error correction at cluster level, DPARSF: Data Processing Assistant for Resting-State fMRI, FDR: false discovery rate, FSL: FMRIB Software Library, HC: healthy controls, MDD: major depressive disorder, REST: REsting State fMRI data analysis Toolkit, RFT: random field theory, TFCE: threshold-free cluster enhancement.
